# Impairment of Ribosome Maturation or Function Confers Salt Resistance on *Escherichia coli* Cells

**DOI:** 10.1371/journal.pone.0065747

**Published:** 2013-05-31

**Authors:** Yoichi Hase, Takefusa Tarusawa, Akira Muto, Hyouta Himeno

**Affiliations:** 1 Department of Biochemistry and Molecular Biology, Faculty of Agriculture and Life Science, Hirosaki University, Hirosaki, Japan; 2 RNA Research Center, Hirosaki University, Hirosaki, Japan; University of Lethbridge, Canada

## Abstract

We found that loss of integrity of the ribosome by removal of a putative ribosome maturation factor or a ribosomal protein conferred salt tolerance on *Escherichia coli* cells. Some protein synthesis inhibitors including kasugamycin and chloramphenicol also had a similar effect, although kasugamycin affected neither 16S rRNA maturation nor subunit association into a 70S ribosome. Thus, salt tolerance is a common feature of cells in which maturation or function of the ribosome is impaired. In these cells, premature induction of an alternative sigma factor, σ^E^, by salt stress was observed. These results suggest the existence of a yet-unknown stress response pathway mediated by the bacterial ribosome.

## Introduction

The prokaryotic ribosome is a ribonucleoprotein particle consisting of a large (50S) subunit and a small (30S) subunit. The 50S subunit, which consists of 23S rRNA, 5S rRNA and over 30 proteins, has a peptidyl transferase center, three tRNA binding sites and a GTPase-associated center. The 30S subunit, which consists of 16S rRNA and over 20 proteins, facilitates the initiation processes of translation and is involved in decoding the genetic message and controlling the fidelity of the codon-anticodon interaction. The maturation of ribosomes are highly elaborate processes, involving cleavage and trimming of the precursors of rRNA, modifications of ribosomal proteins and rRNA, ordered binding of ribosomal proteins and sequential conformational changes [1], [2]. These processes take approximately 2 min and require the help of a considerable number of non-ribosomal factors *in vivo*, although a functional ribosomal particle can be reconstituted from its components *in vitro* without any non-ribosomal factors [3], [4], [5].

RsgA (ribosome small subunit-dependent GTPase A, also known as YjeQ in *Escherichia coli* or YloQ in *Bacillus subtilis*) is a putative ribosome maturation factor having a GTPase activity that is activated by the 30S subunit [6], [7]. RsgA binds around the tRNA binding sites of the 30S subunit [8] to release RbfA, another ribosome maturation factor, from the 30S subunit at nearly the last stage of the ribosome biosynthesis [9]. Deletion of the gene for RsgA from the *E. coli* genome results in slow cell growth, accumulation of 17S RNA, which is a typical precursor of 16S rRNA, and a decreased level of subunit assembly of the ribosome [7], [10].

Our previous study showed that removal or inactivation of RsgA conferred salt tolerance on *E. coli* cells [11]. Defects in processing into 16S rRNA and ribosome assembly in RsgA-deletion cells were restored by salt stress, although the 70S ribosomes are dissociated into subunits in wild-type cells transiently after salt shock. Osmotic shock by upshift of salt or sugar concentration in the culture medium results in some physical changes in *E. coli* cells, such as dehydration and shrinkage of cells [12], which induce uptake of potassium ions and efflux of putrescine within a few minutes of osmotic upshift so that potassium ion replaces putrescine as a nucleic acid counterion [13], [14]. Subsequently, the cell begins to synthesize or uptake osmoprotectants such as glycine betaine, proline and trehalose, while inhibiting general σ^70^ transcription [15]. In consideration of such a drastic change in the physiological condition inside the cell after salt shock, it is possible that RsgA disturbs ribosome maturation under a high salt stress condition, although it usually promotes maturation.

In the present study, we found that removal of other ribosome-associated factors including RbfA or of a ribosomal protein also provides *E. coli* cells with resistance to high salt stress, indicating that salt tolerance is a common feature of cells possessing an increased level of impaired ribosomes. Increased salt tolerance was also provided by treatment of cells with some protein synthesis inhibitors including kasugamycin and chloramphenicol. This is the first report to show that a chemical substance provides cells with salt tolerance. Furthermore, high salt stress prematurely induced an alternative sigma factor, σ^E^, which has a role in maintaining the cell envelope integrity under various stress conditions [16], in mutant cells in which maturation or function of the ribosome is impaired. These results suggest the presence of a novel stress response pathway mediated by the bacterial ribosome.

## Materials and Methods

### *E. coli* strains

BW25113 (genotype, Δ*(araD-araB)567*, Δ*lacZ4787*(::*rrnB*-3), lambda^−^, *rph-1*, Δ*(rhaD-rhaB)568*, *hsdR514*) was used as wild-type. BW25113 *rsgA::kan* (Δ*rsgA*), BW25113 *rimM::kan* (Δ*rimM*), BW25113 *rpsF::kan* (Δ*rpsF*), and BW25113 *rrmJ::kan* (Δ*rrmJ*) were derived from the Keio collection [17]. BW25113 *rbfA::kan* (Δ*rbfA*), an intermediate strain for construction of W3110 *rbfA::kan* [9], was a gift from Dr. Simon Goto.

### Culture conditions

*E. coli* BW25113 or its derivatives were cultured at 37°C with shaking at 130 rpm in LB medium with or without additional NaCl for salt shock. Culture was usually performed in 100 ml of medium in a 300 ml flask. In our previous study, 1.0 M NaCl was added to the culture of *E. coli* W3110 derivatives for salt shock [11], while we used 0.9 M NaCl for the *E. coli* BW25113 derivatives, as these have a lower resistance to salt shocks than W3110 derivatives.

### Sedimentation profile

Cells were ruptured with the same weight of alumina powder, and the cell debris was removed by centrifugation at 4°C for 20 min at 15,000 g. The extract was loaded on a 5–20% (w/v) sucrose density gradient containing 10 mM Tris-HCl (pH 7.8), 10 mM MgCl_2_, 60 mM NH_4_Cl and 1 mM DTT, and it was centrifuged at 36,000 rpm using a P40ST rotor (Hitachi) for 3 hours at 4°C. The gradient was fractioned and the ribosomal concentration of each fraction determined by measuring the absorption at 260 nm (A_260_).

### Preparation of RNA

RNA was extracted from cells with phenol containing 1% SDS and subjected to ethanol precipitation. The resulting RNA fraction was incubated with RNase-free DNase and was subjected to a second round of phenol extraction and ethanol precipitation. This procedure (DNA treatment, phenol extraction and ethanol precipitation) was repeated twice and the concentration of RNA was determined by measuring the absorption at 260 nm (A_260_).

### qRT-PCR

cDNA was prepared from an identical quantity (500 ng) of total RNA using Molony Murine Leukemia Virus (Takara) and a reverse primer corresponding to each gene (see below). The resulting cDNA was subjected to PCR reaction using a DyNAmo HS SYBR Green qPCR Kit (FINNZYMES) and an appropriate set of gene-specific primers (*rpoE* forward, 5′-ACCAGGTCCTGGTTGAACGG-3′; *rpoE* reverse, 5′-GCATAAAGTGGCGAGTCTGG-3′; *rpoH* forward, 5′-ATGACTGACAAAATGCAAAG-3′; *rpoH* reverse, 5′-AGCGCCCGCTCCTCGTCAGC-3′; *MicA* forward, 5′-CGCATTTGTTATCATCATCC-3′; *MicA* forward, 5′-GAAAAAGGCCACTCGTGAG-3′). Reactions were conducted using the DNA Engine OPTICON 2 Continuous Fluorescence Detection system (MJ Research). After initial heating of the sample for 10 min at 95°C, 35 cycles were performed, starting with 10 sec at 95°C, followed by 50°C for 10 sec and finally 72°C for 20 sec. The difference in cycle threshold between samples was calculated using a program attached to the machine. The identities of PCR products were examined by electrophoresis, and the uniformity of the amplified DNA was checked by its melting curve using a program attached to the machine.

## Results

### *E. coli* cells defective in ribosome maturation have tolerance to high salt stress

**Figure 1:**
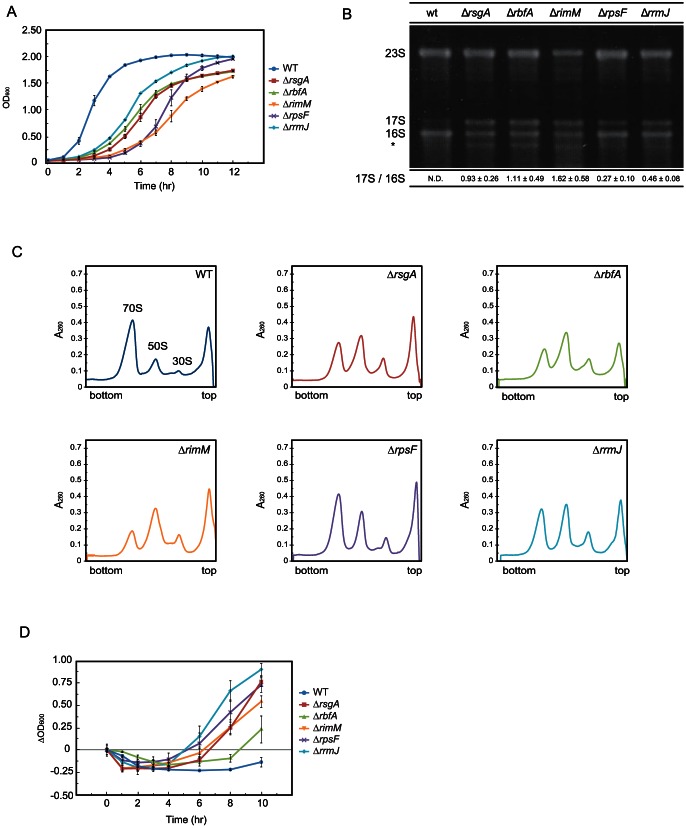
Properties of mutant cells and effect of salt shock on cell growth. (A) Growth of *E. coli* mutant cells under normal culture conditions. Growth of wild-type, ▵*rsgA*, ▵*rbfA*, ▵*rimM*, ▵*rpsF* or ▵*rrmJ* cells at 37°C in LB medium was monitored by measuring OD_600_. (B) Accumulation of 17S RNA in mutant cells. One µg of total RNA fraction prepared from each of the cells was electrophoresed on 1.8% agarose gel. The 3′ truncation product of 16S rRNA is indicated by an asterisk [11]. The band of slightly lower migration than that of 16S rRNA was confirmed as 17S RNA by northern hybridization (Figure S1). The ratio of the amount of 17S RNA to that of 16S rRNA is shown below each lane. (C) Defect in subunit assembly of ribosome in mutant cells. Cells were lysed with alumina powder and the cell debris was removed as described in Materials and Methods. Ten A_260_ units of crude cell extracts were fractionated by 5%–20% sucrose density gradient ultracentrifugation. (D) Growth curves of mutant cells after salt shock. Cells were grown at 37°C in LB medium. When OD_600_ had reached 0.8, 0.9 M NaCl was added to the medium. The OD_600_ value subtracted from that measured immediately after salt shock is plotted.

In a previous study, we found that removal of RsgA conferred salt tolerance on *E. coli* W3110 cells [11]. Salt tolerance was also provided by removal of RsgA in *E. coli* BW25113 cells (Figure 1A). To investigate whether the salt resistance is a specific phenotype to an RsgA-deleted strain, we examined the effect of salt stress on growth of an *E. coli* strain lacking a gene for another putative maturation factor for the 30S subunit, *rbfA* [18] or *rimM* [19]. RsgA, RbfA and RimM play their roles in maturation of the 30S subunit at different timings: first RimM, then RbfA, and last RsgA [8], [20]. We also focused on the effects of deletion of *rpsF* encoding the ribosomal protein S6 [21], which is available from the Keio collection, and *rrmJ*, a gene for methyltransferase (RrmJ) that catalyzes the 2′-*O*-methylation of the ribose at U2552 of 23S rRNA [22].

Under normal growth conditions, deletion of any of these genes from the *E. coli* genome leads to slow cell growth (Figure 1A). We next examined the property of the ribosomes in each cell. As reported previously [18], [19], [20], deletion of *rbfA* or *rimM* results in a considerable level of accumulation of 17S RNA, a typical precursor of 16S rRNA (Figure 1B, Figure S1). We found that 17S RNA also accumulates in Δ*rpsF* and Δ*rrmJ*. The level of 17S RNA that accumulates in Δ*rbfA* or Δ*rimM* is almost comparable to that of Δ*rsgA*. In Δ*rpsF* and Δ*rrmJ*, the level of 17S RNA is slightly lower than that in Δ*rsgA*. We also found that the ratio of the level of 70S ribosomes to that of the 50S or 30S subunits is decreased in cells with deletion of each of these genes (Figure 1C).

The effect of salt shock on growth rate of each strain was then examined (Figure 1D). The cells were cultivated in LB medium, and 0.9 M NaCl was added when the OD_600_ value reached 0.8. As reported previously, Δ*rsgA* cells stop growing immediately after salt shock but begin to grow efficiently from a few hours after salt shock, while the growth of wild-type cells remains stopped until ten hours after salt shock. Interestingly, all mutant strains used have tolerance to salt stress. Δ*rpsF* and Δ*rrmJ* shows prominent tolerance with a degree almost comparable to that of Δ*rsgA* strain. Δ*rbfA* and Δ*rimM* grow slightly but reproducibly faster than wild-type cells.

We then analyzed the ribosome profile in mutants gaining salt tolerance. As shown in Figure 1, the levels of dissociated subunits relative to 70S ribosomes in Δ*rsgA*, Δ*rbfA*, Δ*rimM*, Δ*rpsF* or Δ*rrmJ* cells are significantly higher than that in wild type cells before salt shock. At four hours after salt shock, the level of 70S ribosomes decreased and instead dissociated subunits accumulate in these mutant cells as well as in wild type cells (Figure S2). At eight hours after salt shock, the level of 70S ribosomes relative to the dissociated subunits is increased (Figure S2). This situation is in contrast to that of the wild type cells in which considerable level of 70S ribosomes remain dissociated until eight hours after salt shock.

These results indicate that salt tolerance is conferred by removal of not only RsgA but also other proteins involved in maturation of the 30S subunit, and even a maturation factor for the 50S subunit or a ribosomal protein (S6). Note that the protein products derived from these genes for deletion used in this study bind various regions of the 70S ribosome: RsgA and RbfA bind the tRNA binding sites around helix 44 in the body [8], [23], [24], [25], while RimM binds the head of the 30S subunit [20]. S6 bind to the platform of the 30S subunit [26]. RrmJ binds the A-loop in the 50S subunit [27]. In addition, the composition of ribosomal proteins in the immature 30S subunits varies depending on cells used, which has been checked by quantitative mass spectroscopy [20] or Tris-Tricine PAGE (Goto S., unpublished results). This suggests that salt resistance is provided by impairment of the ribosome maturation regardless of the stage of maturation. We also found that not every single knock-out does result in an increased salt tolerance (Figure S3). For example, deletion of *ksgA* encoding KsgA, a methyltransferase that modifies A1518 and A1519 in the 3'-terminal helix 45 of the 16S rRNA [28], has little or no effect on salt tolerance. Unlike Δ*rsgA*, Δ*rbfA*, Δ*rimM*, Δ*rpsF* and Δ*rrmJ*, Δ*ksgA* shows almost normal cell growth, subunit pattern of ribosomes and processing of 16S rRNA in the absence of salt shock [29].

### Some ribosome-targeting antibiotics provide cells with salt tolerance

**Figure 2:**
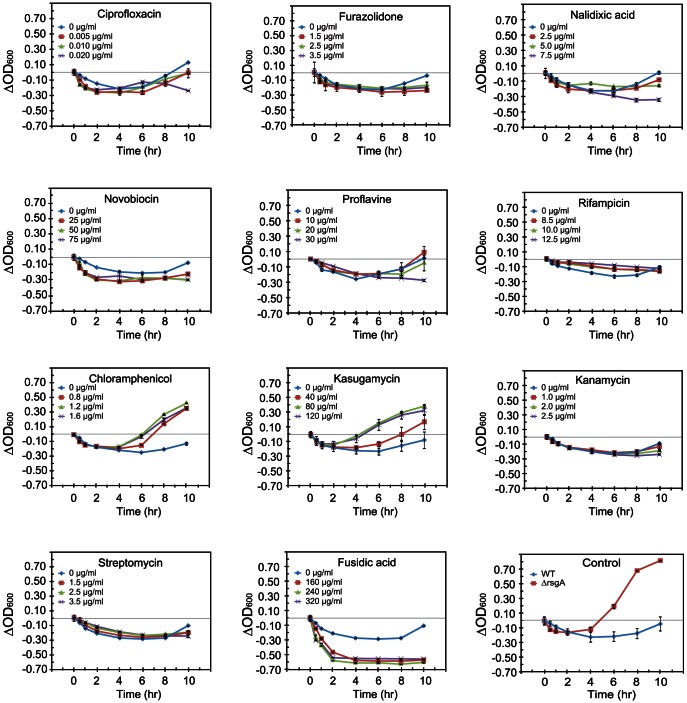
Effects of inhibitors of DNA synthesis, transcription and translation on salt tolerance. Wild type cells were grown at 37°C in LB medium with the indicated concentration of each drug. When OD_600_ reached 0.8, 0.9 M NaCl was added to the medium. Three different concentrations for each antibiotic, which were determined in consideration of the inhibitory effect on the growth of wild type cells in LB medium in the absence of salt shock, were used (Table S1). Growth of drug-untreated ▵*rsgA* cells is shown as the control.

The above results suggest that the salt tolerance of a cell can be induced by a defect in ribosome maturation or ribosome function. Ribosomal function can also be impaired by the addition of antibiotics. We next examined the effects of several antibiotics including inhibitors of DNA synthesis, transcription and translation, on salt tolerance of wild-type cells (Figure 2, Table S1). Wild-type cells were grown in LB medium containing an antibiotic, and cells were subjected to salt shock when OD_600_ had reached 0.8.

Inhibitors of DNA synthesis, ciprofloxacin, furazolidone, nalidixic acid, novobiocin and proflavine, have negative effects on salt tolerance of cells. Cells treated with rifampicin, a transcription inhibitor, have no resistance to salt stress. In contrast, some protein synthesis inhibitors, kasugamycin and chloramphenicol, have pronounced effects. The growth rate of kasugamycin-treated cells after salt shock is significantly higher than that of cells not treated with kasugamycin. Kasugamycin mimics the codon nucleotides at the P- and E-sites by binding within the mRNA path to indirectly inhibit binding of P-site tRNA during the translation initiation process [30]. Chloramphenicol, which inhibits the peptidyl transferase activity for the elongation process of translation [31], [32], also has a similar effect. At 8 hours after salt shock, the ratio of the levels of 70S ribosomes to the dissociated subunits in cells treated with kasugamycin or chloramphenicol is significantly higher than that in cells untreated with antibiotics (Figure S2). Other protein synthesis inhibitors, kanamycin and streptomycin, which bind around the A-site to cause miscoding [33], and fusidic acid, which inhibits the turnover of EF-G [34], have no significant effect on salt tolerance of cells.

### Kasugamycin had no effect on processing into 16S rRNA or subunit association into a 70S ribosome

**Figure 3:**
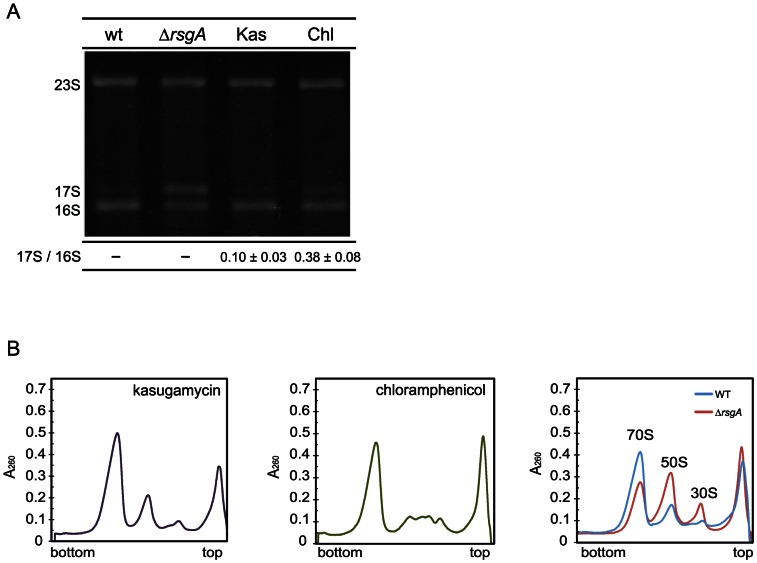
Effects of protein synthesis inhibitors on processing of 17S RNA and on subunit association. Wild-type cells were grown at 37°C in LB medium with 80 µg/ml kasugamycin (Kas) or 1.2 µg/ml chloramphenicol (Chl). (A) One µg of total RNA fraction prepared from wild-type, ▵*rsgA*, or drug-treated cells was electrophoresed on 1.8% agarose gel. The ratio of the amount of 17S RNA to that of 16S rRNA is shown below each lane. (B) Ribosome profiles of wild-type cells, RsgA-deletion cells and wild-type cells treated with protein synthesis inhibitors are shown. Cells were lysed with alumina powder and the cell debris was removed as described in Materials and Methods. Ten A_260_ units of crude cell extracts were fractionated by 5%–20% sucrose density gradient ultracentrifugation.

To investigate whether the observed salt tolerance by some protein synthesis inhibitors is due to a defect in ribosome maturation, the effects of antibiotics, which conferred pronounced salt tolerance on the cells, on ribosome maturation were examined (Figure 3). We analyzed total RNA prepared from these cells by gel electrophoresis to study the degree of processing of 17S RNA into 16S rRNA (Figure 3A). As shown previously, 17S RNA accumulates in *rsgA*-deleted cells [7]. Accumulation of 17S RNA is also detected in chloramphenicol-treated cells, although in a lesser extent [7], and this may be caused by unbalanced synthesis of ribosomal proteins [35]. In contrast, little 17S RNA accumulates in kasugamycin-treated cells as in wild-type cells.

We also examined the pattern of ribosomal subunits from drug-treated cells by sucrose density gradient sedimentation for comparison with that from non-treated cells (Figure 3B). As observed previously [7], the majority of 70S ribosomes are dissociated into subunits in Δ*rsgA* cells. In contrast, kasugamycin or chloramphenicol has only a small effect on subunit association.

These results indicate that the increased salt tolerance of cells does not necessarily require defects in processing into 16S rRNA or subunit association of ribosome. The increased salt tolerance of *E. coli* cells can be attributed to a defect either in ribosome maturation or in some process of translation.

### Salt stress induced premature activation of σ^E^ in salt-resistant cells

The key pathway involved in adaptation to salt stress is control of the outer membrane protein expression. In *E. coli,* an alternative sigma factor, σ^E^, encoded by the *rpoE* gene plays a central role in maintaining the cell envelope integrity [16], [36]. σ^E^ is usually sequestered in the cytoplasmic membrane by an anti-sigma factor, RseA, while it is released from RseA into the cytoplasm by exocytoplasmic stresses including salt stress. Then activated σ^E^ regulates the expression of periplasmic and outer membrane proteins, and it also positively regulates transcription of stress-related factors including σ^E^ itself [37], [38]. We therefore investigated the expression patterns of *rpoE* encoding σ^E^ under a salt stress condition by qRT-PCR.

**Figure 4:**
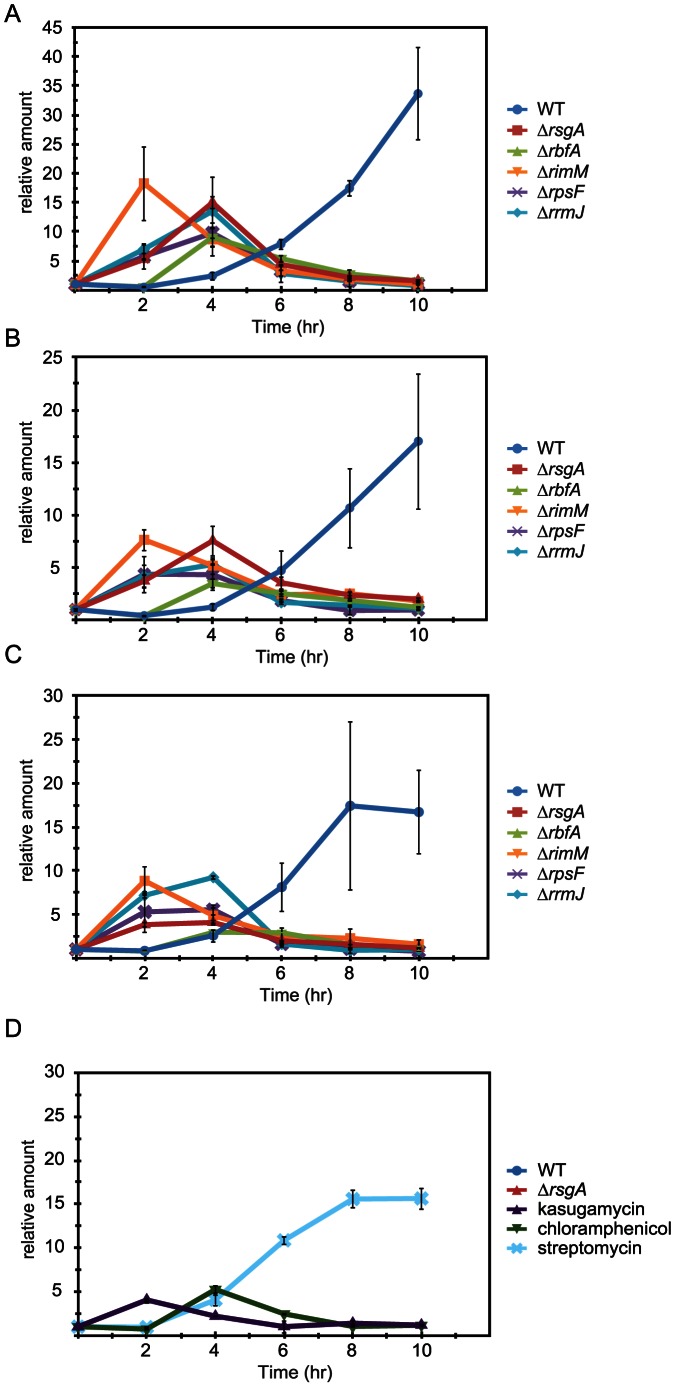
Premature and transient induction of genes regulated by σ^E^. Cells in which a ribosome maturation factor or a ribosomal protein was removed (A–C) and wild-type cells treated with 80 µg/ml kasugamycin, 1.2 µg/ml chloramphenicol or 2.5 µg/ml streptomycin (D) were used. Levels of *rpoE* mRNA (A and D), *rpoH* mRNA (B) and MicA (C) at the indicated time points after salt shock were analyzed by qRT-PCR. Total RNA fraction prepared from each of the cells was used as the template.

It has been shown that 0.464 M sucrose induces *rpoE* within 30 minutes [39]. Consistently, the level of *rpoE* mRNA in either wild-type or Δ*rsgA* cells is immediately elevated after a moderate level of salt shock by the addition of 0.6 M NaCl and it returned to the initial level within one hour (data not shown). It was found that a higher level of salt shock (0.9 M NaCl) used in the present study delays the timing of induction. The level of *rpoE* mRNA begins to increase from about four hours after salt shock and continues to increase until at least 8 hours in wild-type cells (Figure 4A). Intriguingly, the timing of induction in Δ*rsgA* cells is a few hours earlier than that in wild-type cells. The level of *rpoE* mRNA begins to increase about one hour after salt shock and then decreases from 4 or 5 hours after salt shock.

We also investigated the expression patterns of other RNAs regulated by σ^E^, *rpoH* mRNA encoding a sigma factor, σ^32^, involved in heat shock response [40], and MicA, a small non-coding RNA that downregulates the expression of the gene for an outer membrane protein, OmpA, at the translational level [41], [42], [43]. In each mutant cell used in this study, the levels of these RNAs begin to increase about one hour after salt shock, but then decrease from 4 or 5 hours after salt shock (Figure 4B, 4C). This is in sharp contrast to the expression levels of these RNAs in wild-type cells, in which they begin to increase from about four hours after salt shock and continues to increase until at least 8 hours. Such expression patterns of *rpoH* and *micA* in wild-type and mutant cells are similar to those of *rpoE*.

Premature and transient expression of *rpoE* mRNA is also observed in wild-type cells treated with kasugamycin or chloramphenicol but not in those treated with streptomycin, which has no detectable effect on salt tolerance (Figure 4D).

## Discussion

The present study demonstrates increased salt tolerance of several *E. coli* variants lacking either a ribosome maturation factor (RsgA, RbfA, RimM or RrmJ) or a ribosomal protein S6. Salt tolerance is provided regardless of the site of action of the depleted protein on the 70S ribosome and of the stage of maturation of the immature 30S subunits accumulated, although it is apparently involved in some impairment of the ribosomes. These results suggest that increased salt tolerance of cells should be attributed to a general mechanism involved in the ribosome rather than a specific event by a specific ribosome maturation factor. Furthermore, some kinds of translation inhibitors, kasugamycin and chloramphenicol, also provide cells with salt tolerance. Kasugamycin accumulates no detectable level of immature ribosomes, suggesting that the salt tolerance is caused not only by impairment in the ribosome maturation.

Osmotic shock by upshift of salt induces several physical changes in *E. coli* cells, such as dehydration and shrinkage of cells [44], which are sensed by osmosensors embedded in the cytoplasmic membrane, inducing uptake of potassium ions and efflux of putrescine within a few minutes so that potassium ion replaces putrescine as a nucleic acid counterion [45], [46]. Concomitantly, the cell begins to accumulate potassium glutamate in the cell. It induces a σ^S^-dependent gene expression to uptake osmoprotectants such as glycine betaine, proline and trehalose, which counteract the osmotic pressure for survival of cell, while down-regulating general σ^70^ transcription [47]. How ribosome impairment is involved in this pathway has yet to be studied. In the present study, we found that any impairment of ribosome by removal of a ribosome-associated factor or by drug treatment induces premature activation of σ^E^. σ^E^ may contribute to salt tolerance such that it reinforces the outer membrane protein expression. For example, it induces MicA that down-regulates the expression of the gene for an outer membrane protein, OmpA, at the translational level. Earlier induction of σ^E^ would contribute to salt tolerance to some extent, considering that σ^E^ regulates expression of outer membrane proteins and that it induces a heat shock sigma factor. Because induction of σ^E^ appears to begin from a few hours after salt shock, the effects of impairment of the ribosome function on earlier events should be focused in the future study.

As far as we know, increased tolerance of cells to osmotic stress by removal of a ribosome maturation factor or addition of a chemical substance has never been reported. Or rather, even increased sensitivity to osmotic stress by removal of a ribosome maturation factor has been reported in *Saccharomyces cerevisiae* [48]. In mammalian cells, impaired ribosome biogenesis induces a p53-dependent stress response pathway [49]. Some kinds of ribosome-targeting antibiotics including kanamycin and streptomycin induce heat shock proteins in *E. coli*, and other kinds of those antibiotics including chloramphenicol, fusidic acid and tetracycline induce cold shock proteins, leading to the proposal that bacterial ribosome acts as a sensor of temperature for the control of global regulatory networks [50]. The present results strongly suggest the presence of a novel stress response pathway, which is mediated by the bacterial ribosome. For example, the following pathway can be assumed: some quantitative and/or qualitative perturbation in protein synthesis activity by the loss of integrity of ribosome or its function affects homeostasis of the cell membrane, causing premature induction of σ^E^ to confer salt tolerance on cells.

## Supporting Information

Figure S1 **Accumulation of 17S RNA in Δ** ***rsgA*** **, Δ** ***rbfA*** **, Δ** ***rimM*** **, Δ** ***rpsF*** **or Δ** ***rrmJ*** **cells.** One mg of total RNA was subjected to electrophoresis on denaturing MOPS-formaldehyde 1.8% agarose gel. The band of slightly lower migration than that of 16S rRNA was confirmed as 17S RNA by northern hybridization using 3′-DIG labeled probe 9 and probe 12, which are complimentary to 16S rRNA and its 5′ extension, respectively (Hase *et al*., 2009).
(EPS)

Figure S2 **Effects of depletion of a maturation factor or a ribosomal protein S6 and addition of protein synthesis inhibitor on subunit association of the ribosome.** Cells depleted of a maturation factor or S6 were grown at 37°C in LB medium, and wild type cells were grown at 37°C in LB medium supplemented with 80 µg/ml kasugamycin or 1.2 µg/ml chloramphenicol. When OD_600_ reached 0.8, 0.9 M NaCl was added to the medium. Cells, which were collected at 0, 4 and 8 hours after salt shock, were lysed with alumina powder and the cell debris was removed as described in Materials and Methods. Crude cell extracts were fractionated by 5%–20% sucrose density gradient ultracentrifugation. Peak height of each subunit relative to that of 70S ribosome is shown above the peak.
(EPS)

Figure S3 **Effect of salt shock on cell growth of Δ** ***ksgA*** **or Δ** ***lonA*** **.** (A) Growth of wild-type, Δ*rsgA*, Δ*ksgA* or Δ*lonA* cells at 37°C in LB medium was monitored by measuring OD_600_. (B) Cells were grown at 37°C in LB medium. When OD_600_ had reached 0.8, 0.9 M NaCl was added to the medium. The OD_600_ value subtracted from that measured immediately after salt shock is plotted. (C) Accumulation of 17S RNA in Δ*ksgA* and Δ*lonA* cells. One µg of total RNA fraction prepared from each of the cells was electrophoresed on 1.8% agarose gel. The 3′ truncation product of 16S rRNA is indicated by an asterisk.
(EPS)

Table S1 **Concentrations of drugs used in this study.** Three different concentrations for each antibiotic, which were determined in consideration of the inhibitory effect on the growth of wild type cells in LB medium in the absence of salt shock, were used. The lowest of the three concentrations had almost no effect on growth rate. The highest concentration was determined so that it inhibited the growth rate of wild type cells in the absence of salt stress to the level similar to that of Δ*rsgA* cells. Some antibiotics such as kanamycin and streptomycin drastically decreased the plateau level of growth curve, and in this case the highest concentration was adjusted so that OD_600_ at the plateau was nearly equal to or slightly lower than 1.0. At each time point, OD_600_ was measured and compared with that of wild type cells grown without antibiotics.
(DOCX)
