# Supplementary material for: Impairment of Ribosome Maturation or Function Confers Salt Resistance on Escherichia coli Cells
Source: PLoS One. 2013 May 31;8(5):e65747. doi: 10.1371/journal.pone.0065747 (PMC3669203; doi:10.1371/journal.pone.0065747)
Supplement: Table S1 — Concentrations of drugs used in this study. Three different concentrations for each antibiotic, which were determined in consideration of the inhibitory effect on the growth of wild type cells in LB medium in the absence of salt shock, were used. The lowest of the three concentrations had almost no effect on growth rate. The highest concentration was determined so that it inhibited the growth rate of wild type cells in the absence of salt stress to the level similar to that of ΔrsgA cells. Some antibiotics such as kanamycin and streptomycin drastically decreased the plateau level of growth curve, and in this case the highest concentration was adjusted so that OD600 at the plateau was nearly equal to or slightly lower than 1.0. At each time point, OD600 was measured and compared with that of wild type cells grown without antibiotics. (DOCX) [file pone.0065747.s004.docx]

Table S1.

-------------------------------------------------------------------------------------------------------

Concentration of drug (µg/ml)

-----------------------------------------------------------------------------

Name of drug lowest medium highest

-------------------------------------------------------------------------------------------------------

Ciprofloxacin 0.005 0.010 0.020

Furazolidone 1.5 2.5 3.5

Nalidixic acid 2.5 5 7.5

Novobiocin 25 50 75

Proflavine 10 20 30

Rifampicin 8.5 10 12.5

Chloramphenicol 0.8 1.2 1.6

Kasugamycin 40 80 120

Kanamycin 1 2 2.5

Streptomycin 1.5 2.5 3.5

Fusidic acid 160 240 320

-------------------------------------------------------------------------------------------------------
